# Association of vitamin D deficiency with hepatitis B virus - related liver diseases

**DOI:** 10.1186/s12879-016-1836-0

**Published:** 2016-09-23

**Authors:** Nghiem Xuan Hoan, Nguyen Khuyen, Mai Thanh Binh, Dao Phuong Giang, Hoang Van Tong, Phan Quoc Hoan, Ngo Tat Trung, Do Tuan Anh, Nguyen Linh Toan, Christian G. Meyer, Peter G. Kremsner, Thirumalaisamy P. Velavan, Le Huu Song

**Affiliations:** 1 Institute of Tropical Medicine, University of Tübingen, Tübingen, Germany; 2 Vietnamese-German Center for Medical Research (VG-CARE), Hanoi, Vietnam; 3 Department of Infectious Diseases, Duc Giang Hospital, Hanoi, Vietnam; 4 Department of Molecular Biology, 108 Military Central Hospital, Hanoi, Vietnam; 5 Department of Infectious Diseases, Vietnam Military Medical University, Hanoi, Vietnam; 6 Department of Pathophysiology, Vietnam Military Medical University, Hanoi, Vietnam; 7 Fondation Congolaise pour la Recherche Medicale, Brazzaville, Republic of Congo; 8 Institute of Clinical Infectious Diseases, 108 Military Central Hospital, Tran Hung Dao Street N1, Hai Ba Trung District, Hanoi, Vietnam

**Keywords:** Vitamin D deficiency, HBV infection, Chronic liver disease, Liver cirrhosis, Hepatocellular carcinoma

## Abstract

**Background:**

As an immune modulator, vitamin D is involved in various pathophysiological mechanisms in a plethora of diseases. This study aims to correlate the vitamin D deficiency status and clinical progression of liver diseases associated with hepatitis B virus (HBV) infection in patients in Vietnam and to compare it to healthy controls.

**Methods:**

We quantified the levels of total vitamin D [25-(OH) D2 and D3] in serum samples from 400 HBV patients (chronic hepatitis B infection [CHB], *n* = 165; HBV-associated liver cirrhosis [LC], *n* = 127; HBV-associated hepatocellular carcinoma [HCC], *n* = 108) and 122 unrelated healthy controls (HC). Univariate and multivariate analyses were performed in order to determine the association between vitamin D levels and distinct clinical parameters.

**Results:**

The prevalence of vitamin D inadequacy (<30 ng/mL) was high among healthy individuals (81.7 %) as well as in HBV patients (84.3 %). Vitamin D deficiency (<20 ng/ml) or severe deficiency (<10 ng/ml) was observed more frequently among HBV patients (52 %) and subgroups (CHB, 47.8 %; LC, 54.4 %; HCC, 55.3 %) compared to the control group (32.5 %) (*P* < 0.001). Vitamin D levels and HBV-DNA load were strongly and inversely correlated (rho = −0.57, *P* < 0.0001). Multivariate regression analysis also revealed an independent association of HBV-DNA loads with low vitamin D levels (*P* = 0.0004). In addition, reduced vitamin D levels were associated with significant clinical progression of LC (Child-Pugh C versus Child-Pugh A, *P* = 0.0018; Child-Pugh C versus Child-Pugh B, *P* = 0.016).

**Conclusions:**

Vitamin D deficiency was observed in the majority of HBV-infected patients and associated with adverse clinical outcomes. Our findings suggest that substitution of vitamin D may be a supportive option in the treatment of chronic liver diseases, in particular of HBV-associated disorders.

## Background

Hepatitis B virus (HBV) infection is a major health problem which may be life-threatening due to it's frequent severe complications, including fulminant acute hepatitis, liver cirrhosis (LC) and hepatocellular carcinoma (HCC) [1]. Interactions between host immune responses and HBV determine the clinical manifestations of HBV infection. Innate immune mechanisms have important roles in the clearance of HBV through production of inflammatory cytokines such as interferons (IFN-α/β and IFN-γ) [1].

Besides the classical function in regulating calcium and bone homeostasis, vitamin D (cholecalciferol, 25-(OH)D_3_) is also relevant in modulating both innate and adaptive immune responses [2, 3]. Deficiency of vitamin D has been shown to be involved in carcinogenesis and the course of several infectious diseases [2–4], among them distinct liver infections [5–8].

In humans, vitamin D supplementation can result in an increased activity of intrinsic IFN-α signaling [9] and supplementation of vitamin D is a supportive option in the treatment of pulmonary tuberculosis [10, 11]. Natural sources of vitamin D are mostly vitamin D_3_ (cholecalciferol) retrieved from nutrition and sunlight exposure. Vitamin D_2_ is not produced in humans, and small amounts of vitamin D_2_ (ergocalciferol) are derived from edible plants [12]. Both vitamin D_3_ and D_2_ are physiologically inactive. Hydroxylation occurs in the liver, forming the intermediate metabolite (calcidiol, 25-(OH)D). Calcidiol is further converted into the active form (calcitriol, 1,25-(OH)_2_D) in the kidney [13] and released in the circulation. Precise measurement of calcitriol is intricate due to its low serum concentrations and short half-life. Therefore, assessment of serum concentrations of both forms of 25-(OH)D_3_ and D_2_ are required for the diagnosis of vitamin D deficiency [13].

Up to 90 % of vitamin D required for physiological functions is derived from production and supply in the skin, while the remainder is retrieved from the diet [14–16]. Production of vitamin D in the skin significantly depends on sunshine exposure, geographical habitation and skin pigmentation. However, vitamin D deficiency may occur globally. In developed countries, vitamin D deficiency is very common, with almost half of the population being affected [13]. In Vietnam, a previous study has demonstrated that vitamin D deficiency among women was approximately 30 % and, thus, almost twice of that in men (16 %) [17]. The prevalence of HBV infections currently ranges from 10 to 20 % in the Vietnamese population and HBV-related liver diseases are foreseen and predicted to be a notable public health burden in the next decades [18]. The present study aimed to study the association of vitamin D deficiency status with the progression of HBV-related liver diseases, including chronic hepatitis (CHB), liver cirrhosis (LC) and hepatocellular carcinoma (HCC) in Vietnam.

## Methods

### Subjects and sample collection

Four hundred Vietnamese HBV-infected patients were enrolled for this cross-sectional study at the 108 Military Central Hospital, Hanoi, Vietnam, between 2012 and 2014. HBV patients were assigned to the different clinical subgroups based on clinical manifestations. Briefly, CHB patients (*n* = 165) were characterized based upon clinical symptoms such as fatigue, anorexia, jaundice, hepatomegaly, hard density of the liver, splenomegaly, hyperbilirubinemia, elevated levels of AST and ALT and HBsAg positivity for more than 6 months. LC patients (*n* = 127) were characterized as being infected with HBV and presenting clinical manifestations such as anorexia, nausea, vomiting, malaise, weight loss, abdominal distress, jaundice, edema, cutaneous arterial spider angiomas, ascites, shrunken liver, splenomegaly, hyperbilirubinemia, elevated levels of AST and ALT, prolonged serum prothrombin time, and decreased serum albumin. HCC patients (*n* = 108) were characterized as being infected chronically with HBV and presenting abdominal pain, an abdominal mass in the right upper quadrant, blood-tinged ascites, weight loss, anorexia, fatigue, jaundice, prolonged serum prothrombin time, hyperbilirubinemia, elevated levels of AST, ALT and serum a-fetoprotein (AFP), ultrasound showing a tumor, and histopathology indicating tumor cells. None of the patients had a history of alcohol or drug abuse. HBV-DNA loads were measured by RT-PCR using the 7500 Fast Real-Time PCR System (Applied Biosystems, Foster City, California, USA) and liver function tests, including assessment of alanine transaminase (ALT) and aspartate transaminase (AST) enzyme levels, alpha fetoprotein (AFP), platelet count (PLT), total and direct bilirubin, albumin and prothrombin were performed using AU640 Chemistry Analyzer (Beckman Coulter, California, USA). Based on Child-Pugh scores, HBV-related LC patients were categorized as Child-Pugh A, −B and -C [19]. In addition, 122 healthy individuals devoid of HBsAg were randomly enrolled from the hospital’s blood bank as controls. None of these healthy individuals had a history of any chronic liver disease, nor were they immunosuppressed. All participants were confirmed negative for anti-HCV and anti-HIV antibodies by using commercial ELISA assays (Diagnostic automation/Cortez Diagnostics, Inc., Woodland Hills, California, USA).

### Vitamin D measurement

Total vitamin D [25-(OH)D2 and D3] levels were measured in the serum samples from patients and controls using a commercial ELISA kit (Gentaur, Kampenhout, Belgium) according to the manufacturer’s instructions. The kit’s range of vitamin D assessment is 4.39 to 133 ng/ml with a coefficient of variation of 20 %. Based on the recommendation of the Endocrine Society (Maryland, USA; http://www.endocrine.org/) [20], a level of 30 ng/ml or above is considered as vitamin D sufficiency. We, therefore, categorized the vitamin D status as normal (≥30 ng/ml), insufficient (20–29.9 ng/ml), deficient (10–19.9 mg/ml), and severely deficient (<10 ng/ml).

### Statistics

All statistical analyses were performed using R version 3.1.2 (http://www.r-project.org). Chi-square test, Kruskal-Wallis test, and Mann-Whitney-Wilcoxon test were used to compare differences between groups for qualitative or quantitative variables where appropriate. Correlations between variables were evaluated using the Spearman’s rank correlation test. We used univariate analyses, multivariate logistic regression and multivariate linear regression models to test for associations of serum 25-(OH)D_3_ status with clinical parameters and independent risk factors. Bonferroni correction was applied as appropriate for multiple comparisons. The level of significance was set at *P* < 0.05.

## Results

### Patients and control characteristics

The baseline characteristics of the 400 HBV-infected patients and 122 healthy controls are given in Table 1. The majority of both patients and healthy controls were males (85.3 and 67.2 %, respectively). As expected, the median age of patients increased with progression of liver disease. Healthy controls were significantly younger than patients (*P* < 0.001). The levels of ALT and AST were significantly higher in patients with CHB compared to those with LC and HCC (ALT: *P* < 0.001 and AST: *P* = 0.014). As also expected, albumin and prothrombin levels as well as platelet counts were significantly lower in patients with LC compared to those without LC (*P* < 0.01). AFP levels were significantly higher among the HCC group compared to the CHB and LC groups (*P* < 0.001).

**Table 1:** Characteristics of the study cohort

Characteristics	CHB (*n* = 165)	LC (*n* = 127)	HCC (*n* = 108)	HC (*n* = 122)	*P* value
Age (median, range)	39 (18–79)	55 (19–84)	55 (18–81)	40 (19–58)	<0.001
Gender (male/female)	135/30	107/20	99/9	82/40	<0.001
Liver cirrhosis stage:					
Child-Pugh A (*n*, %)	NA	42 (33.0 %)	NA	NA	NA
Child-Pugh B (*n*, %)	NA	62 (48.8 %)	NA	NA	NA
Child-Pugh C (*n*, %)	NA	23 (18.2 %)	NA	NA	NA
WBC (×10^3^/ml)	6.5 (3.6–13.9)	6.1 (1.8–20.5)	5.2 (2.1–15.1)	NA	0.007
RBC (×10^6^/ml)	4.9 (2.5–6.6)	3.6 (1.9–6.4)	4.2 (2.4–7.2)	NA	<0.001
PLT (×10^3^/ml)	197.5 (21.5–472)	76.5 (24.4–641)	161 (6.7–428)	NA	<0.001
AST (IU/L)	100 (14–7700)	93 (26–1221)	53 (18–653)	NA	0.014
ALT (IU/L)	120 (9–4908)	51.5 (8–1000)	43 (4–401)	NA	<0.001
Total-Bilirubin (mg/dl)	15.3 (6–499)	35.5 (6.4–733)	18 (9–185)	NA	<0.01
Direct-Bilirubin (mg/dl)	4.5 (1–300)	14.7 (1.6–449)	5.6 (1.1–156)	NA	<0.01
Albumin (g/L)	42 (16–53)	30 (16–54)	39.4 (22–47)	NA	<0.001
Prothrombin (%)	89 (41–140)	53.5 (14–101)	77.8 (20–127)	NA	<0.001
HBV-DNA (log10 copies/ml)	5.6 (2.4–10.1)	NA	NA	NA	NA
Alpha fetoprotein (IU/L)	2.7 (1.6–320)	9.2 (1.5–300)	196 (1.3–400)	NA	<0.001

*Abbreviation*: *CHB* chronic hepatitis B, *LC* liver cirrhosis, *HCC* hepatocellular carcinoma, *HC* healthy control, *WBC* white blood cells, *RBC* red blood cells, *PLT* platelets. *AST* and *ALT* aspartate and alanine amino transferase, *IU* international unit, *NA* not applicable. Values given are medians and range. *P* values were calculated by Chi-square test and Kruskal-Wallis test where appropriate

### Serum vitamin D levels in HBV infected patients and in controls

**Fig. 1:**
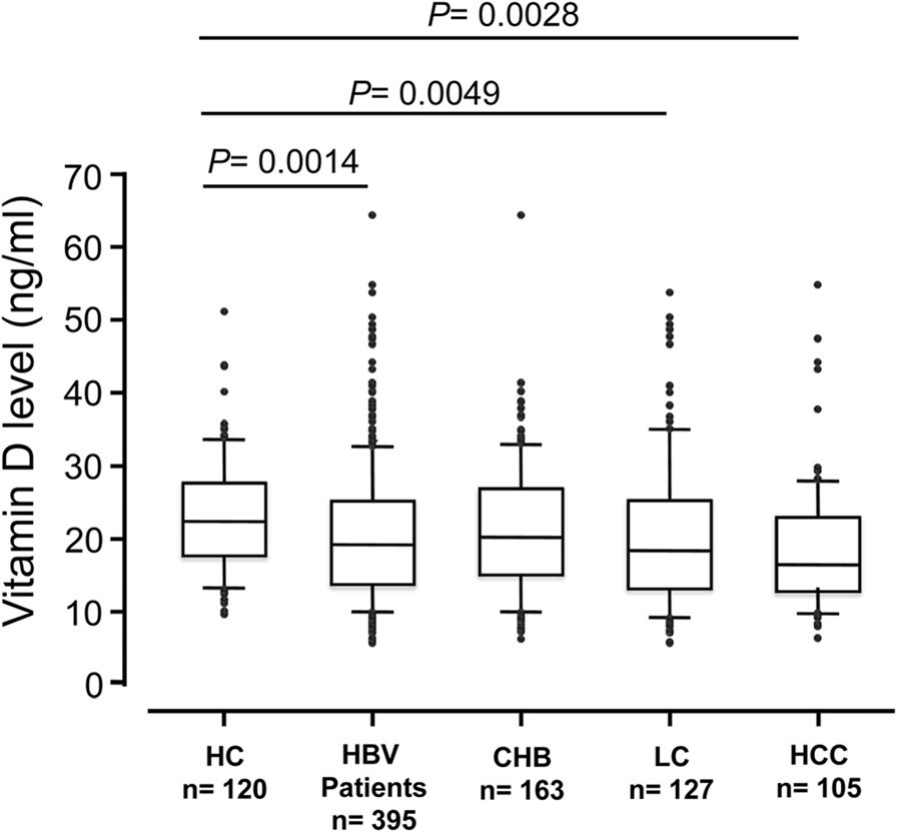
Vitamin D levels in healthy individuals (HC), HBV patients and subgroups. Chronic hepatitis B (CHB), liver cirrhosis (LC) and hepatocellular carcinoma (HCC); *P* values were calculated by Mann-Whitney Wilcoxon test

**Fig. 2:**
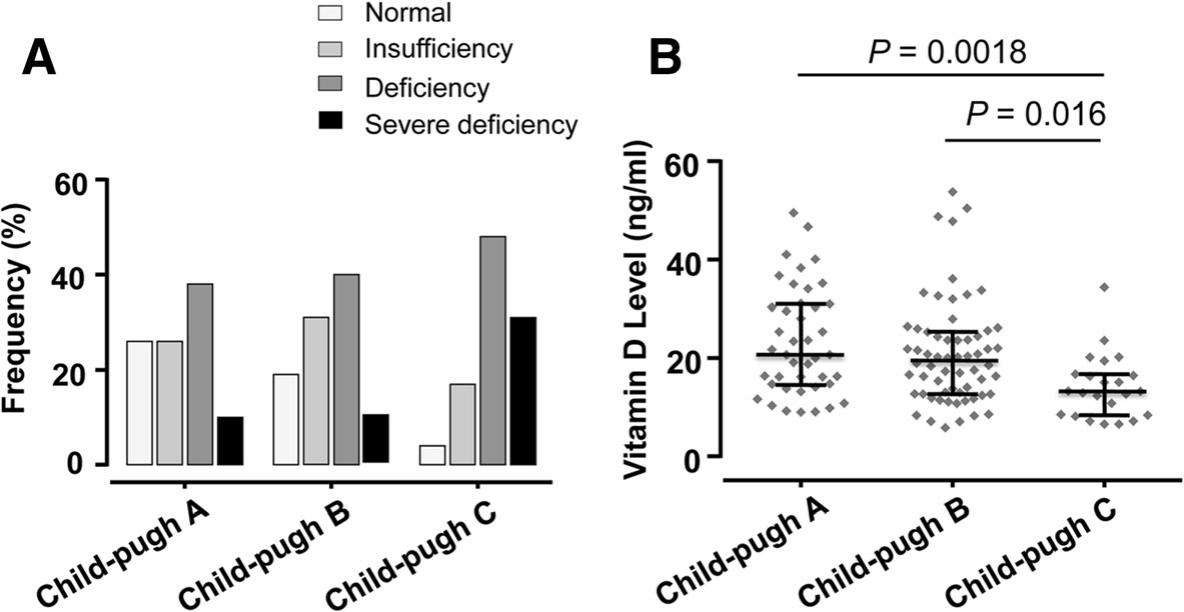
Distribution of vitamin D levels in HBV related liver cirrhosis patients. **a** Vitamin D levels in different stages of HBV-related liver cirrhosis according to Child-Pugh classification. **b** Vitamin D levels in different stages of HBV-related liver cirrhosis. *Dot-plots* illustrate medians with interquartile ranges. *P* values were calculated by Mann-Whitney-Wilcoxon test

Seven individuals, each two from the HC and the CHB groups, and three from the HCC group had undetectable vitamin D levels (<4.39 ng/ml). Vitamin D levels were mostly inadequate (<30 ng/ml) in both HBV patients (84.3 %) and controls (81.7 %); the difference, however, was not significant. When vitamin D levels were stratified into the four categories of normal levels (≥30 ng/ml), insufficiency (20–29.9 ng/ml), deficiency (10–19.9 mg/ml), and severe deficiency (<10 ng/ml), deficiency and severe deficiency occurred frequently among all HBV patients (51.9 %) and in all subgroups compared to healthy controls (32.5 %) (*P* < 0.001) (Table 2). Vitamin D levels were 20.7 ± 9.2 ng/ml in HBV-infected patients and 23.6 ± 9.5 ng/ml in HCs. In the patient subgroups, vitamin D levels were 21.2 ± 8.9 ng/ml in CHB, 20.6 ± 10.4 ng/ml in LC, and 20 ± 8.3 ng/ml in HCC. Vitamin D levels were higher in HCs than in HBV patients (HBV patients vs. HC: *P* = 0.0014; LC vs. HC: *P* = 0.0049; HCC vs. HC: *P* = 0.0028) (Fig. 1). Among the HBV patients, vitamin D levels were decreased with progression of HBV-related liver diseases. However, significant differences were not observed when comparing the subgroups (CHB vs. LC, CHB vs. HCC and LC vs. HCC) (Fig. 1). Deficiency and severe vitamin D deficiency were significantly associated with advanced LC according to Child-Pugh classifications (Fig. 2a and b). Of 127 LC patients, 30.4 % patients of the Child-Pugh C group had severe deficiency, while 10 % only in the Child-Pugh A and B groups had severe deficiency (Fig. 2a). In addition, vitamin D levels were significantly lower in Child-Pugh C patients (15.1 ± 6.5 ng/ml), compared to Child-Pugh B (20.8 ± 10.6 ng/ml) and Child-Pugh A patients (23.3 ± 10.7 ng/ml) (*P* = 0.0018 and *P* = 0.016, respectively) (Fig. 2b).

**Table 2:** Distribution of HBV-infected patients and healthy controls with Vitamin D status

Vitamin D stratum	HC (*n* = 120)	HBV patients (*n* = 395)	CHB (*n* = 163)	LC (*n* = 127)	HCC (*n* = 105)
	*n* (%)	*n* (%)	*n* (%)	*n* (%)	*n* (%)
Normal	22 (18.3)	62 (15.7)	29 (17.8)	24 (18.9)	9 (8.5)
Insufficiency	59 (49.2)	128 (32.4)	56 (34.3)	34 (26.7)	38 (36.2)
Deficiency	38 (31.7)	164 (41.5)	61 (37.4)	52 (41.0)	51 (48.6)
Severe deficiency	1 (0.8)	41 (10.4)	17 (10.4)	17 (13.4)	7 (6.7)

*CHB* chronic hepatitis B, *LC* liver cirrhosis, *HCC* hepatocellular carcinoma, *HC* healthy control, *HBV* hepatitis B virus

*P* = 0.00024 (Chi-square test for all groups including HC, CHB, LC, and HCC)

*P* = 0.00016 (Chi-square test: HBV patients vs. HC)

### Factors associated with vitamin D levels and vitamin D deficiency

**Fig. 3:**
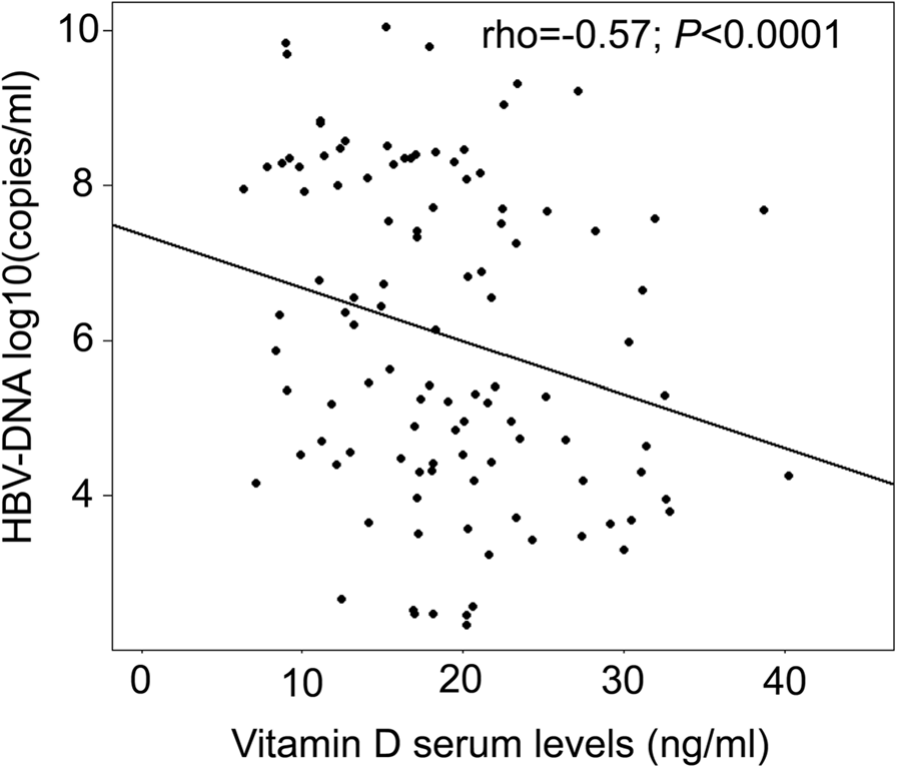
Correlation between Vitamin D levels with HBV-DNA loads. The correlation between vitamin D levels and HBV-DNA loads in chronic hepatitis B patients was calculated by using Spearman’s rank correlation coefficient. Spearman’s rho (rho) and *P* value are given

Of 165 CHB patients, 63 only were available for analyses of the correlation between vitamin D levels and HBV-DNA loads. Vitamin D levels and HBV-DNA were significantly and inversely correlated (rho = −0.57, *P* < 0.0001) (Fig. 3). Other clinical parameters were either not or weakly correlated with vitamin D serum levels. This applied to the PLT (rho = 0.05), ALT (rho = 0.16), AST (rho = 0.20), total-bilirubin (rho = 0.03), direct-bilirubin (rho = 0.04), albumin (rho = 0.002), AFP (rho = −0.02) and prothrombin levels (rho = 0.07) (data not shown). We also performed multivariate linear regression models to determine the independent factors correlated with vitamin D levels. Only HBV-DNA loads were independently and inversely correlated with vitamin D levels (*P* = 0.0004) (Table 3).

**Table 3:** Factors associated with baseline vitamin D levels in HBV patients

Variables	Univariate analysis	Multivariate analysis	
	*P* value	*P* value	Coefficient β
Age (continuous)	0.47	0.44	−0.07
Gender (male vs. female)	0.4	0.87	−0.49
HBeAg (positive vs. negative)	0.105	0.11	5.26
ALT levels (continuous)	0.08	0.51	0.008
HCC vs. non-HCC	0.019	0.13	7.5
Cirrhosis vs. non-cirrhosis	0.47	0.61	1.4
HBV-DNA (log10 copies/ml, continuous)	<0.0001	0.0004	−1.9

Univariate analysis and multivariate linear regression model for independent factors were used to correlate vitamin D levels with clinical parameters. *HBeAg* Hepatitis B envelope antigen, *ALT* alanine amino transferase, *HCC* hepatocellular carcinoma

Next, we applied univariate analysis and multivariate logistic regression models to assess the determinants associated with vitamin D deficiency. HBV infection and HBeAg negativity were independently associated with low vitamin D levels (<30 ng/ml) in both univariate and multivariate analyses. HCC was also a risk factor for low vitamin D levels in the univariate analysis (Table 4). Other independent factors such as age, gender, and the occurrence of LC were not associated with any degree of vitamin D deficiency.

**Table 4:** Univariate and multivariate logistic regression analyses of factors associated with vitamin D deficiency

Variables	Univariate analysis		Multivariate analysis	
	OR (95 % CI)	*P* value	OR (95 % CI)	*P* value
HBV versus non-HBV (or HC) ^†^	1.2 (0.7–2.0)	0.49	10.8 (1.4–81.8)	0.021
HCC versus non-HCC ^‡^	2.4 (1.1–5.0)	0.029	2.4 (0.3–21.6)	0.43
Cirrhosis versus non-cirrhosis ^β^	1.4 (0.8–2.5)	0.23	1.2 (0.5–2.7)	0.74
HBeAg (negative versus positive) ^γ^	3.6 (1.2–10.7)	0.015	3.8 (1.2–12.0)	0.024

Univariate and multivariate logistic regression model for independent risk factors were applied. Independent risk factors added into the multivariate logistic regression model are age, gender, HCC status, cirrhosis status and HBeAg status. (†), 395 HBV cases vs. 120 HC; (‡), 105 HCC cases vs. 290 non-HCC HBV patients; (β), 127 LC patients vs. 268 non-cirrhosis HBV patients; (γ), 266 HBeAg negative vs. 129 HBeAg positive patients. *HBV* hepatitis B virus, *HC* healthy control, *HBeAg* Hepatitis B envelope antigen, *HCC* hepatocellular carcinoma

## Discussion

Vitamin D has not only important functions in the metabolism of calcium and bone homeostasis but also manifold effects in the fine regulation of immune responses [2, 3, 13]. There is increasing evidence of vitamin D deficiency effects on a wide spectrum of diseases, including osteoporosis, autoimmunity, asthma, infectious diseases, several forms of malignancy and even psychiatric disorders [13, 21, 22]. This study shows that insufficiency of vitamin D occurs more frequently among healthy individuals as well as HBV patients. Vitamin D levels were inversely correlated with HBV-DNA loads and were associated with more severe conditions of LC patients. These findings suggest that serum vitamin D levels contribute significantly to the clinical courses of HBV infection, including the severe consequences of LC and HCC.

A high prevalence of vitamin D deficiency (>90 %) in chronic liver disease has been reported to be associated with liver disease progression [23–25]. High prevalence of inadequate vitamin D status, as observed in both healthy populations and in patients with HBV-related liver diseases, indicate that low vitamin D levels are frequent, along with osteoporosis in populations of many geographical regions [13, 26]. Roughly, one billion people worldwide apparently are vitamin D deficient [13, 27]. Vitamin D deficiency occurs rather among the elderly population [28, 29] and is more frequent in women [13, 17]. In this study, the predominance of males in all study groups and the significant difference in age between cases and healthy individuals might influence the degree of vitamin D deficiency between cases and healthy individuals. In the multivariate analyses adjusted for age and gender, HBV infection appears to be an independent risk factor for vitamin D inadequacy that augments the risk for osteoporosis and other vitamin D deficiency-related diseases. The high prevalence of HBV infection in Vietnam (10–20 %) [18] is an important factor favoring the occurrence of osteoporosis and many other diseases related to vitamin D deficiency.

In this study, deficiency and severe vitamin D deficiency were observed more frequently in patients with HBV-related liver disease and were found significantly associated with the end-stage of liver cirrhosis (Child-Pugh C). Vitamin D is involved in inhibition of inflammation and abrogation of liver fibrosis, substantiated by the observation that vitamin D receptor knockout mice spontaneously develop hepatic fibrosis [30, 31]. Apparently, vitamin D deficiency is a cause of fibrosis progression [6, 8, 32]. The interaction between vitamin D and its receptor can modulate the immune response involved in the pathogenesis of many solid cancers [33, 34]. Our study is in line with previous studies which have indicated an association of vitamin D levels with HCC [35–38]. Vitamin D can inhibit the growth of HCC cell lines both in vitro and in vivo [39], and supplementation of vitamin D and calcium has substantially reduced the risk of cancer in a randomized clinical trial [40]. In addition, vitamin D analogs and vitamin D receptor activators such as maxacalcitol (OCT), 16-ene analogs, 19-nor analogs, LG190119 and C-20 cyclopropylcal-citriol have been developed and tested in a pre-clinical study [41–43]. Vitamin D analogs and VDR activators may in the future be promising adjuvants in HCC treatment.

The causes of vitamin D deficiency in chronic liver disease are considered multi-factorial [13, 21]. An explanation for the low vitamin D levels observed in chronic liver disease could be an impaired liver function. Hepatic injuries lead to decreased production of vitamin D carrier proteins such as vitamin D binding protein and albumin, and impaired hepatic hydroxylation of vitamin D to calcidiol or 25-(OH)D in the liver. Vitamin D levels were shown to be positively correlated with albumin levels and platelet counts and inversely correlated with ALT levels during the active phase of chronic liver disease [6, 24, 25]. Serum vitamin D levels of <10 ng/ml can be a predictive factor for low serum albumin levels and the severity of chronic liver disease [32, 44]. In the present study, however, vitamin D inadequacy was not significantly correlated with liver function parameters, possibly due to the fact that vitamin D serum levels are affected by multiple factors and most of the patients were not in the active phase during sample collection.

In line with a previous study [45], our results corroborate a strongly inverse correlation between HBV-DNA loads and vitamin D levels. We assume that vitamin D deficiency, as frequently observed in HBV infection, can fail to suppress HBV replication. A link between vitamin D deficiency and susceptibility to various infections and the development of various inflammatory diseases has been suggested [2, 3]. In addition, low vitamin D levels linked to low sustained virological response to interferon-based therapy in chronic hepatitis B and C have been demonstrated in clinical studies [8, 25]. Although vitamin D deficiency is associated with adverse clinical outcomes of HBV infection, our study has limitations. Serum vitamin D levels are affected by several factors, including seasonal variation, sunshine exposure, geographical habitation and diet. However, any information concerning the season at the time of sampling and diet of the study subjects was not available. Another limitation is that the study was a cross-sectional study and, thus, we could not determine the fluctuation of vitamin D levels over the course of HBV infection as well as the causative association of vitamin D levels and HBV-related liver diseases. Vitamin D may be an additional factor for the clinical outcomes of HBV infection. The influence of vitamin D metabolism on HBV-related liver diseases remains unclear and the role of vitamin D and its interaction with vitamin D receptor on the pathogenesis of HBV infection needs to be explored further.

## Conclusions

Vitamin D deficiency was observed in the majority of HBV patients particularly in advanced liver diseases and associated with adverse clinical outcomes. Our findings allow speculating that vitamin D and its analogs might provide a potential therapeutic addendum in the treatment of chronic liver diseases.

## Abbreviations

AFP: Alpha feto protein
AST and ALT: Aspartate and alanine amino transferase
CHB: Chronic hepatitis B
HBV: Hepatitis B virus
HC: Healthy control
HCC: Hepatocellular carcinoma
LC: Liver cirrhosis
PLT: Platelets
